# GlnK Regulates the Type III Secretion System by Modulating NtrB-NtrC Homeostasis in *Pseudomonas aeruginosa*

**DOI:** 10.3390/microorganisms14020339

**Published:** 2026-02-02

**Authors:** Xiaomeng Sun, Qitong Du, Yiming Li, Xuetao Gong, Yu Zhang, Yongxin Jin, Shouguang Jin, Weihui Wu

**Affiliations:** State Key Laboratory of Medicinal Chemical Biology, Key Laboratory of Molecular Microbiology and Technology of the Ministry of Education, Department of Microbiology, College of Life Sciences, Nankai University, Tianjin 300071, China; 1120220629@mail.nankai.edu.cn (X.S.);

**Keywords:** *Pseudomonas aeruginosa*, GlnK, type III secretion system, NtrB-NtrC, lung infection

## Abstract

Bacterial pathogens exploit host-derived nutrients to coordinate metabolism and virulence determinants to optimize fitness in vivo. In *Pseudomonas aeruginosa*, GlnK is a central regulator of nitrogen metabolism. It senses the intracellular nitrogen status by integrating 2-oxoglutarate (2-OG) and glutamine signals, which in turn triggers its uridylylation and conformational changes. This reversible post-translational modification modulates its interaction with target proteins, thereby precisely regulating carbon-nitrogen metabolic homeostasis and enabling adaptive nitrogen metabolism in response to host-derived nutrient cues. In this study, we found that *glnK* is upregulated during infection in a mouse pneumonia model. By growing bacteria in mouse bronchoalveolar lavage fluid (BALF), we demonstrated that the expression of *glnK* is activated by the NtrB-NtrC two-component regulatory system in response to the host nutrient environment. Mutation of *glnK* impairs bacterial virulence. Transcriptomic analysis revealed downregulation of the type III secretion system (T3SS) genes in the *glnK* mutant. Further studies revealed a role of GlnK in maintaining the homeostasis of the NtrB-NtrC system through a negative feedback mechanism, which is required for the expression of the T3SS genes. Collectively, these findings reveal a role of GlnK in interconnecting carbon–nitrogen balance and the T3SS in response to the host environment.

## 1. Introduction

*Pseudomonas aeruginosa* is an opportunistic Gram-negative pathogen that poses substantial threats to human health, particularly in immunocompromised populations, individuals with cystic fibrosis and severe burns, and those suffering from chronic wounds [1,2,3]. A key driver of its pathogenicity lies in its ability to survive and proliferate within hosts. This exceptional adaptability is attributed to its capacity to sense and integrate environmental cues, such as fluctuations in nutrient availability, which in turn allows it to optimize metabolic pathways and virulence-associated traits [4,5].

The type III secretion system (T3SS) is one of the key virulence factors of *P. aeruginosa* [6]. This specialized syringe-like apparatus directly delivers effector proteins into host cells to disrupt immune responses and promote colonization [7]. Transcription of the T3SS genes is controlled by the master regulator ExsA [8]. The *exsA* gene is located in the *exsCEBA* operon and driven by the P*_exsC_* promoter and an adjacent P*_exsA_* promoter that is located in the intergenic region between *exsB* and *exsA*. The P*_exsA_* promoter is primarily regulated by cAMP in partnership with the cAMP receptor protein (CRP) Vfr [9]. Under T3SS-non-inducing conditions, ExsA is sequestered by ExsD and ExsC binds to ExsE. Under T3SS-inducing conditions, e.g., Ca^2+^ depletion or contact with host cells, the intracellular cAMP level is increased, resulting in activation of *exsA* transcription from the P*_exsA_* promoter [10,11]. Meanwhile, ExsE is secreted through the T3SS machinery, releasing ExsC to sequester ExsD, resulting in free ExsA that directly activates the expression of T3SS machinery and effector genes [12]. The P*_exsC_* promoter is also activated by ExsA, thus forming a positive feedback regulation [13].

Previous studies have demonstrated that during lung infection, long-chain fatty acids (LCFAs) and mucin are major carbon sources of *P. aeruginosa* [14,15]. We found that a regulator, PvrA, senses the LCFA metabolic intermediate molecule palmitoyl coenzyme A and regulates genes involved in LCFA metabolism and the PQS quorum sensing system [16].

Besides carbon sources, nitrogen is indispensable for microbial survival. Bacteria predominantly utilize reactive forms of nitrogen such as ammonium (NH_4_^+^) and nitrate (NO_3_^−^) [17]. NO_3_^−^ is usually converted into the preferred ammonium (NH_4_^+^), and ammonium assimilation is mediated by two specialized pathways. The high-affinity GS-GOGAT pathway proceeds in two steps: glutamine synthetase (GS) first assimilates ammonium into glutamine (GLN), and GLN then condenses with α-ketogluterate (α-KG) via glutamate synthase (GOGAT) to generate glutamate. In contrast, the glutamate dehydrogenase (GDH) pathway relies on GDH to directly catalyze the reductive amination of α-KG with ammonium into glutamate. This pathway functions efficiently only when ammonium is abundant [18]. Together, glutamate and glutamine act as versatile nitrogen donors in transamination and transamidation reactions, supporting microbial metabolism and growth.

Bacterial PII signal transduction proteins play an essential role in sustaining intracellular pools of glutamate and glutamine as well as maintaining cellular carbon–nitrogen balance in response to fluctuating nitrogen levels [19,20]. In *E.coli*, GlnK modulates GS activity to coordinate nitrogen uptake and assimilation in a unified regulatory network. Under nitrogen-replete conditions, GlnK undergoes deuridylylation, adopting a conformation that binds to the bifunctional enzyme adenylyl-transferase/adenylyl-removase (AT/AR) and promotes its adenylyl-transferase activity, leading to GS inactivation via adenylylation (GS~AMP) to prevent excessive nitrogen assimilation. Conversely, under nitrogen-limitation conditions, uridylylated GlnK (GlnK~UMP) reduces the affinity for AT/AR, allowing AT/AR to switch to unadenylylase activity and reactivate GS through deadenylylation [21]. GlnK also regulates AmtB, a high-affinity ammonium transporter responsible for NH_4_^+^ uptake in a nitrogen-dependent manner. Under nitrogen repletion, deuridylylated GlnK binds to AmtB at the cytoplasmic interface, inducing a conformational change that blocks its transport channel and shuts down NH_4_^+^ uptake. Under nitrogen limitation, GlnK~UMP dissociates from AmtB, restoring the activity of the transporter to scavenge scarce environmental NH_4_^+^ [22,23]. This dual regulation of GS and AmtB by GlnK ensures tight coupling of nitrogen acquisition and utilization, optimizing resource allocation and avoiding energy waste on superfluous nitrogen processes. Notably, these interactions are governed by the modification and demodification events of GlnK, a process regulated by the signal-transducing bifunctional uridylyltransferase/uridylyl-removing enzyme (UTase/UR) GlnD. GlnD switches its dual catalytic activity in response to metabolic signals of 2-oxoglutarate (2-OG) and glutamine, with these metabolites also directly influencing GlnK’s conformational dynamics and its subsequent protein–protein interaction affinity. Under nitrogen-replete conditions with high glutamine and low 2-OG, GlnD exerts uridylyl-removing (UR) activity to mediate the deuridylylation of GlnK, whereas under nitrogen-limiting conditions characterized by low glutamine and high 2-OG, it triggers uridylyltransferase (UTase) activity to drive the covalent uridylylation of GlnK. Thus, the GlnD-catalyzed reversible modification of GlnK, together with its direct metabolic modulation, alters its conformational state and ultimately governs the coordinated regulation of glutamine synthetase (GS) activity and ammonium uptake to maintain cellular carbon-nitrogen homeostasis.

In addition to its well-characterized role in regulating GS and AmtB, GlnK also interacts with and modulates the activity of a diverse set of proteins, including various enzymes, transcriptional regulators, and nutrient transporters [24,25]. Most of these interacting proteins participate in the homeostasis of metabolism, highlighting the function of GlnK as a major metabolic hub that coordinates nitrogen metabolism and downstream physiological processes. It has been demonstrated that bacterial metabolism is tightly coupled to pathogenicity [26]. However, whether GlnK is involved in bacterial response to the host environment and the regulation of virulence factors in *P. aeruginosa* remains largely unknown, leaving a critical knowledge gap that warrants further investigation.

In this study, we utilized a murine pneumonia model to investigate the role of GlnK in the pathogenesis of *P. aeruginosa*. We found that the expression of GlnK is increased in response to in vivo nitrogen-limiting conditions. In addition, GlnK contributes to bacterial virulence through the homeostatic regulation of the NtrB-NtrC two-component system, which is required for T3SS activation. These findings reveal that GlnK integrates nitrogen signals with virulence regulation, highlighting nutrient–virulence cross-talk in *P. aeruginosa*.

## 2. Materials and Methods

### 2.1. Bacterial Strains, Plasmids, and Culture Media

The bacterial strains and plasmids employed in this study, along with their descriptions and sources, are summarized in Table S3 [27]. For the cultivation of bacterial cells, Luria–Bertani (LB) medium (5 g/L yeast extract, 10 g/L tryptone, and 5 g/L NaCl, pH 7.4) and M9 minimal medium (22.0 mM KH_2_PO_4_, 2.3 mM Na_2_HPO_4,_ 8.6 mM NaCl, 1 mM MgSO_4_, 0.1 mM CaCl_2_, 18.7 mM NH_4_Cl, and 22.2 mM glucose) were used. Bronchoalveolar lavage fluid (BALF) was collected from healthy 6-week-old female BALB/c mice as previously described [28]. The trachea was exposed and intubated with an 18-GN polyethylene catheter (BD Angiocath™ IV Catheter), followed by instillation with 1 mL sterile 0.9% NaCl. Then the lavage fluid was gently aspirated. The instillation and aspiration was performed twice. The collected BALF samples were sterilized by filtering through a 0.22 μm filter and pooled for further experiments. To ensure experimental consistency, the concentration of each batch of BALF was normalized based on total protein concentration, which was quantified using a BCA Protein Assay Kit (Beyotime, Haimen, China).

### 2.2. Ethics Statement

All animal studies were conducted in accordance with national regulations and the ethical guidelines for animal research formulated by Nankai University. The protocol was approved by the institutional animal care and use committee of the College of Life Sciences of Nankai University (approval code: NK-04-2012; approval date: 12 March 2012).

Specific pathogen-free (SPF) female BALB/c mice, aged 6 weeks and weighing 16–20 g, were purchased from Beijing Vital River Laboratory Animal Technology Co., Ltd. (Beijing, China). The mice were randomly housed in a pathogen-free facility with a maximum of 5 mice per cage. All animals were maintained under a 12 h light and dark cycle at a controlled temperature (20–25 °C).

### 2.3. Mouse Pneumonia Model and Analysis

Indicated bacterial cells were initially cultured in LB at 37 °C overnight. Subsequently, the bacteria were transferred to LB and grown until OD_600_ reached 1.0 (~5 × 10^8^ CFU/mL). The bacterial cells were harvested, washed once with sterile 0.9% NaCl, and then diluted 1:2.5 with sterile 0.9% NaCl adjusted to the concentration of 2 × 10^8^ CFU/mL or maintained at 5 × 10^8^ CFU/mL. For intranasal inoculation, each SPF female BALB/c mouse was anesthetized until loss of the pedal reflex, then placed in a supine position with its head slightly elevated to maintain a patent airway. A 20 μL aliquot of the bacterial suspension was subsequently instilled slowly dropwise into the bilateral nasal cavities (10 μL per nostril), resulting in a final inoculum dose of 4 × 10^6^ CFU or 1 × 10^7^ CFU per mouse. At 12 or 6 h post-infection (hpi), the mice were euthanized via carbon dioxide (CO_2_) inhalation. For bacterial load quantification at 12 hpi, lungs were immediately isolated and homogenized in 1% proteose peptone (Solarbio, Tongzhou, China), and bacterial loads were determined by serial dilution and plating. For histopathological analysis, hematoxylin and eosin (H&E) staining was performed as previously described [29]. Briefly, the lungs were fixed in 4% paraformaldehyde overnight, dehydrated through a graded series, and embedded in paraffin prior to sectioning and H&E staining. For in vivo gene expression analysis at 6 hpi, BALF was collected immediately after euthanasia using an 18-GN polyethylene catheter, via two rounds of sterile 0.9% NaCl injection and aspiration. Following centrifugation, the bacterial pellets were harvested for subsequent RNA extraction.

### 2.4. RNA Extraction and Real-Time Quantitative PCR (RT-qPCR)

Total RNA was isolated using the TransZol Up Plus RNA Kit (TransGen Biotech, Beijing, China) following the manufacturer’s protocol. Prior to washing with the kit’s Wash Buffer, DNase I digestion was performed using RNase-free DNase I (Cat. No. GD201, TransGen Biotech, Beijing, China) to eliminate genomic DNA (gDNA) contamination. After RNA extraction, the concentration of each RNA sample was quantified using a NanoDrop 2000 spectrophotometer (Thermo Fisher Scientific, Waltham, MA, USA), with the A_260_/A_280_ and A_260_/A_230_ ratios used to evaluate RNA purity. Subsequently, complementary DNA (cDNA) was synthesized from 1 μg of total RNA using HiScript III RT SuperMix for qPCR (+gDNA wiper) (Vazyme, Nanjing, China). RT-qPCR was performed using Super Multiple Probe qPCR PreMix (Vazyme, Nanjing, China), alongside no-reverse transcription (no-RT) controls to exclude gDNA contamination. The 30S ribosomal protein gene *rpsL* was employed as the internal reference.

### 2.5. β-Galactosidase Activity Assay

The β-galactosidase activity assay was performed as previously described with minor modifications [29,30]. Bacteria were grown in LB at 37 °C. When the culture reached an OD_600_ of 0.8, 0.1 mM isopropyl β-D-1-thiogalactopyranoside (IPTG) was added to induce NtrC expression in the Δ*ntrC* strain, with parallel identical IPTG induction in the strains of PA14/pMMB67EH and Δ*ntrC*/pMMB67EH. The induction was continued until the OD_600_ reached 1.0. The bacteria were harvested by centrifugation. The cell pellet was resuspended in a buffer containing 0.04 M NaH_2_PO_4_, 0.06 M Na_2_HPO_4_, 0.001 M MgSO_4_, 0.01 M KCl, and 0.05 M β-mercaptoethanol (added freshly prior to use). The OD_600_ of the resuspended bacterial suspension was measured. Subsequently, 10 μL 0.1% (*w*/*v*) sodium dodecyl sulfate (SDS) and 10 μL chloroform were added to 500 μL of the bacterial suspension, followed by vortexing for 10 s to lyse the cells. After adding 100 μL of o-nitrophenyl-β-D-galactopyranoside (ONPG) solution, the mixture was immediately incubated at 37 °C. Once the solution turned pale yellow, the enzymatic reaction was terminated by adding 500 μL of 1M Na_2_CO_3_. The reaction mixture was then centrifuged at 16,000× *g* for 5 min to eliminate cell debris, and the absorbance of the resulting supernatant was measured at OD_420_. The OD_600_ value of each sample was used as the internal control. The calculation formula for Miller units is as follows, where T denotes the reaction duration (min):

$$Miller\;units=\frac{1000\times {OD}_{420}}{500\times T\times {OD}_{600}}$$

### 2.6. Electrophoretic Mobility Shift Assay (EMSA)

The electrophoretic mobility shift assay (EMSA) was performed as previously described with minor modifications [15]. The 59 bp DNA probe was incubated with purified NtrC protein at the indicated concentrations in a 20 μL buffer (100 mM Tris–HCl, pH 7.4, 500 mM KCl, 5 mM EDTA, 35 mM MgCl_2_, and 5 mM DTT). Following incubation, samples were loaded onto a 12% native polyacrylamide gel and electrophoresed at 10 mA for 60 min on ice in 0.5 × TBE (Tris-borate EDTA) buffer (44.5 mM Tris base, 44.5 mM boric acid, and 1 mM EDTA, pH 8.0).

### 2.7. RNA-Seq and Data Analysis

Wild-type PA14 and the Δ*glnK* mutant were cultured in LB at 37 °C to the log phase (OD_600_ = 1.0), followed by RNA purification. The concentration and quality of total RNA from each sample were determined using Agilent 2100/2200 Bioanalyzer (Agilent Technologies, Santa Clara, CA, USA), NanoDrop (Thermo Fisher Scientific Inc., Waltham, MA, USA), and 1% agrose gel electrophoresis. The RNA-seq was performed by Azenta (Suzhou, China). Briefly, starting with 1 μg of total RNA, library construction was completed through rRNA depletion, RNA fragmentation, reverse transcription, end repair, dA-tailing, and adaptor ligation. Subsequently, adaptor-ligated DNA was size-selected to recover ~400 bp fragments and the RNA strand was digested. Libraries were amplified by PCR with indexed P5/P7 primers. The samples were validated by Qsep100 and quantified using Qubit3.0.

Multiplexed libraries were sequenced on an Illumina HiSeq/Novaseq instrument (Illumina, San Diego, CA, USA) employing a 2 × 150 paired-end (PE) mode in accordance with the manufacturer’s instructions. Sequence reads were mapped to the PA14 reference genome (GCF_000014625.1). The DESeq2 Bioconductor package was used for differential expression analysis, with differentially expressed genes identified by a Benjamini–Hochberg-adjusted *p*-value (P_adj_) < 0.05 to control the false discovery rate.

### 2.8. Cytotoxicity Assay

Lactate dehydrogenase (LDH) release assays were employed to assess cellular cytotoxicity. A549 cells were seeded into 24-well plates (2  ×  10^5^ cells per well) and cultured in RPMI 1640 medium supplemented with 10% fetal bovine serum (FBS) at 37 ℃ with 5% CO_2_. Indicated bacterial strains were cultured overnight in LB. On the following day, the bacteria were subcultured into fresh LB and grown to log phase (OD_600_ = 1.0), followed by washing once and resuspension in RPMI 1640 medium. A549 cells were infected with bacteria at a multiplicity of infection (MOI) of 50, followed by centrifugation at 500× *g* for 5 min to synchronize bacterial cell contact. Addition of 1640 medium to parallel wells was used as a blank control. For bacterial strains requiring inducible expression, 1 mM isopropyl β-D-1-thiogalactopyranoside (IPTG) was added to the culture. After 2.5 or 4.5 h of infection, LDH was measured using an LDH Cytotoxicity Assay Kit (Solarbio, Tongzhou, China) following the manufacturer’s instructions. Cells incubated with the kit-supplied LDH release buffer served as the total LDH control group and the cytotoxicity rate was calculated by the following formula:

$$Cytotoxicity\;Rate\left(\%\right)=\left[1-\frac{A_{infected}-A_{blank}}{A_{control}-A_{blank}}\right]\;\times \;100$$

### 2.9. Western Blotting

The protocol was performed as previously described with minor modifications [29]. Equal amounts of bacteria were collected by centrifugation (10,000× *g*, 1 min) and resuspended in 1 × SDS loading buffer. After boiling at 99 °C for 10 min, samples were separated by 12% SDS-PAGE, followed by semi-wet transfer onto polyvinylidene difluoride (PVDF) membrane. The membranes were blocked with 5% non-fat milk in PBST for 1 h at room temperature, incubated with primary antibodies (1:2000 dilution; mouse anti-6 × His, rabbit anti-Flag or mouse anti-RNA polymerase α subunit) for 1 h, washed three times with PBST (7 min each), and then incubated with HRP-conjugated secondary antibodies (1:2000 dilution) for 1 h. Protein bands were detected by an Immobilon Western kit (Millipore, Burlington, MA, USA).

### 2.10. Affinity Chromatography Purification–Mass Spectrometry (AP-MS)

To purify GlnK-His_6_ and GST-His_6_ recombinant proteins, we utilized the Δ*glnK* mutant harboring the pMMB67EH plasmid, on which the fusion protein’s expression was driven by the *tac* promoter. Following growth to OD_600_ 0.5, cells were induced with 1 mM IPTG for 4 h and then harvested by centrifugation (8000× *g*, 10 min). Subsequently, pellets were resuspended in a lysis buffer (300 mM NaCl, 50 mM Na_2_HPO_4_, pH 8.0, and 5 mM imidazole). The cells were sonicated and centrifuged (16,000× *g*, 10 min). The resulting supernatant was incubated with Ni-NTA agarose (QIAGEN, Hilden, Germany) at 4 °C for 2 h with gentle rotation. The resin was washed five column volumes (CVs) with the lysis buffer containing 20 mM imidazole, followed by elution with an elution buffer (pH 8.0) consisting of 300 mM NaCl, 50 mM NaH_2_PO_4_, and 250 mM imidazole.

After purification, the proteins were quantified using a BCA assay. Following enzymatic digestion, equal amounts of protein samples were desalted with C18 reversed-phase solid-phase extraction (RP-SPE) and subjected to a nano-liquid chromatography quadrupole ion trap/orbitrap high-resolution mass spectrometry system (nanoLC-MS/MS) analysis. Protein identification and quantification were performed by matching the acquired peptide fragment spectra against the PA14 reference proteome (derived from genome accession GCF_000014625.1).

### 2.11. Co-Purification Assay

The Δ*glnK* mutant co-expressing GlnK-His_6_ (from the plasmid pMMB67EH) and NtrB-Flag (from the plasmid pAK1900), along with control cells expressing only GlnK-His_6_ (harboring empty pAK1900 plasmid), were cultured in LB. For co-purification analysis, expression of GlnK-His_6_ was induced by IPTG for 4 h. Culture of each strain (25 mL) was harvested by centrifugation (8000× *g*, 10 min). The bacterial pellets were snap-frozen in liquid nitrogen for 30 s before being subjected to Ni-NTA purification [31]. Purified protein samples were analyzed by Western blotting using appropriate antibodies.

### 2.12. Statistical Analysis

Values are shown as mean ± standard deviation (SD). Statistical analyses were conducted with GraphPad software v8.0.1 (San Diego, CA, USA), employing either Student’s *t*-test or ANOVA coupled with Dunnett’s multiple comparison test.

## 3. Results

### 3.1. The glnK Gene Is Upregulated in Response to the Mouse Lung Environment

**Figure 1:**
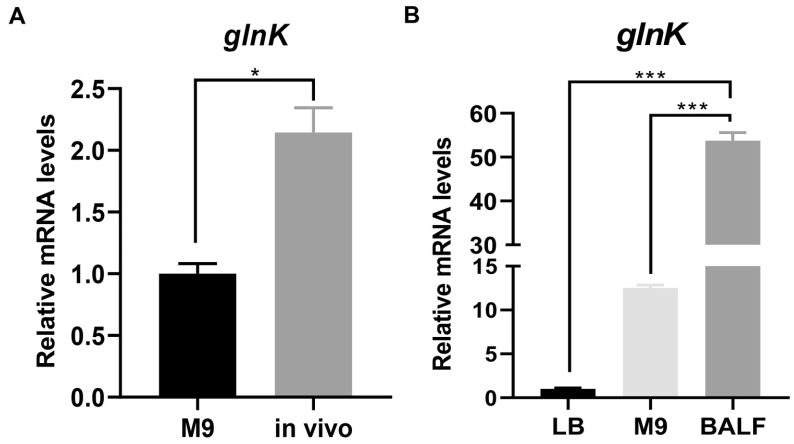
Expression of the *glnK* gene in wild-type PA14 in response to the mouse lung environment. (**A**) mRNA levels of *glnK* in PA14 isolated from mouse lungs at 6 h post-infection, compared to those cultured in M9 minimal medium. * *p* < 0.05 by Student’s *t*-test. (**B**) RT-qPCR analysis of *glnK* mRNA levels in PA14 cultured in BALF, LB, and M9 medium. *** *p* < 0.001 by ANOVA/Dunnett’s multiple comparison test.

To investigate whether GlnK is involved in bacterial response to the host environment, we determined its expression level in a mouse pneumonia model. RT-qPCR results revealed upregulation of *glnK* in bacteria isolated from mouse lungs compared to those grown in LB or M9 medium (Figure 1A and Figure S1). To verify that the nutritional environment of the mouse lung induces the expression of *glnK*, we grew wild-type PA14 in mouse bronchoalveolar lavage fluid (BALF) for 1h, which resulted in upregulation of *glnK* (Figure 1B).

**Figure 2:**
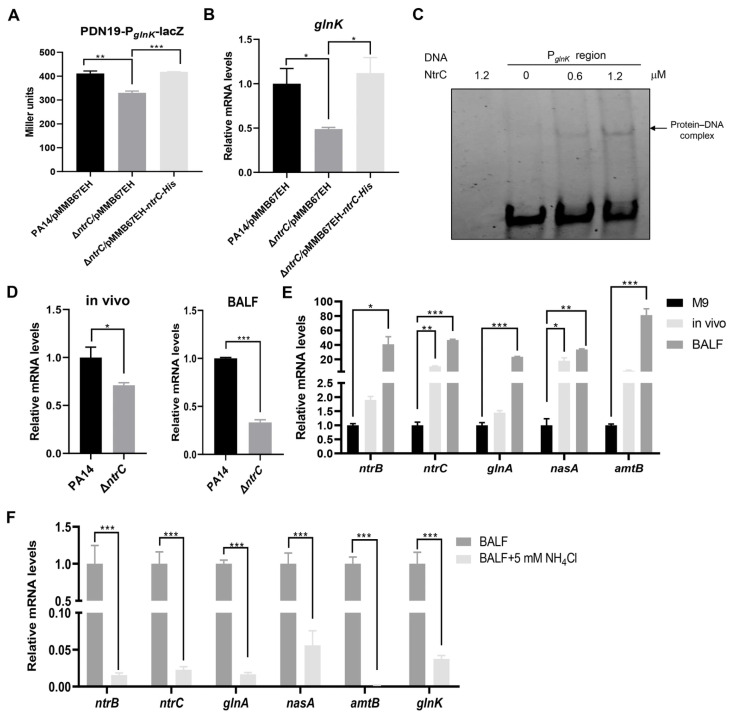
The role  of NtrC in regulating *glnK*. (**A**) PA14, Δ*ntrC*, and the complemented strain (Δ*ntrC*/*ntrC*) harboring the P*_glnK_*-lacZ transcriptional fusion were cultured in LB and induced with 0.1 mM isopropyl β-D-1-thiogalactopyranoside (IPTG) for 15 min. When the OD_600_ reached 1.0, the bacteria were subjected to the β-Galactosidase activity assay. ** *p* < 0.01, *** *p* < 0.001 by ANOVA/Dunnett’s multiple comparison test. (**B**) mRNA levels of *glnK* in the indicated strains. * *p* < 0.05 by ANOVA/Dunnett’s multiple comparison test. (**C**) electrophoretic mobility shift assay (EMSA). The DNA probe corresponds to the *glnK* promoter region (−373 to −318 bp upstream of the start codon). The protein–DNA complex is indicated by an arrow. (**D**) RT-qPCR determination of *glnK* mRNA levels in PA14 and the Δ*ntrC* mutant isolated from mouse lungs or grown in BALF. * *p* < 0.05; *** *p* < 0.001 by Student’s *t*-test. (**E**) mRNA levels of *ntrB*, *ntrC*, and the NtrB-NtrC regulated nitrogen assimilation genes in PA14 cultured in M9 or BALF or isolated from mouse lungs (in vivo). * *p* < 0.05; ** *p* < 0.01; *** *p* < 0.001 by ANOVA/Dunnett’s multiple comparison test. (**F**) mRNA levels of the indicated genes in PA14 cultured in BALF and BALF supplemented with 5 mM NH_4_Cl. *** *p* < 0.001 by Student’s *t*-test.

Then we explored the regulatory mechanism of *glnK*. In *Pseudomonas putida* KT2440, the transcription of *glnK* is directly activated by NtrC in response to nitrogen limitation [32]. Sequence alignment revealed >90% sequence identity between the NtrC ortholog from *P. putida* KT2440 and *P. aeruginosa* PA14 (Figure S2A). Additionally, the *P. putida* NtrC-binding motifs (Motif 1 and Motif 2) are conserved in the *glnK* promoter regions in strains of the two species (Figure S2B) [32]. To investigate the role of NtrC in regulating *glnK* in *P. aeruginosa*, we constructed a *glnK* promoter–lacZ transcriptional fusion. Mutation of *ntrC* reduced LacZ expression, which was restored to wild-type level by complementation with an *ntrC* gene (Figure 2A). Consistently, the *glnK* mRNA level was downregulated in the Δ*ntrC* mutant, which was restored to wild-type level by complementation with an *ntrC* gene (Figure 2B). EMSA results demonstrated the binding of NtrC to the *glnK* promoter region (Figure 2C and Figure S3). In addition, deletion of *ntrC* in wild-type PA14 reduced *glnK* expression in mouse lungs and BALF (Figure 2D). These results demonstrate that *P. aeruginosa* NtrC directly regulates *glnK* in response to the in vivo environment.

The NtrB-NtrC two-component system is activated by nitrogen limitation [33]. Besides *glnK*, the expression of *ntrB*, *ntrC*, and nitrogen assimilation genes regulated by the NtrB-NtrC system was increased in mouse lungs and BALF (Figure 2E). Supplementation of NH_4_Cl in the BALF reduced the expression of these genes and *glnK* (Figure 2F). Collectively, these results demonstrate activation of the NtrB-NtrC system in mouse lungs, which upregulates the expression of *glnK*.

### 3.2. Glnk Regulates Bacterial Virulence

**Figure 3:**
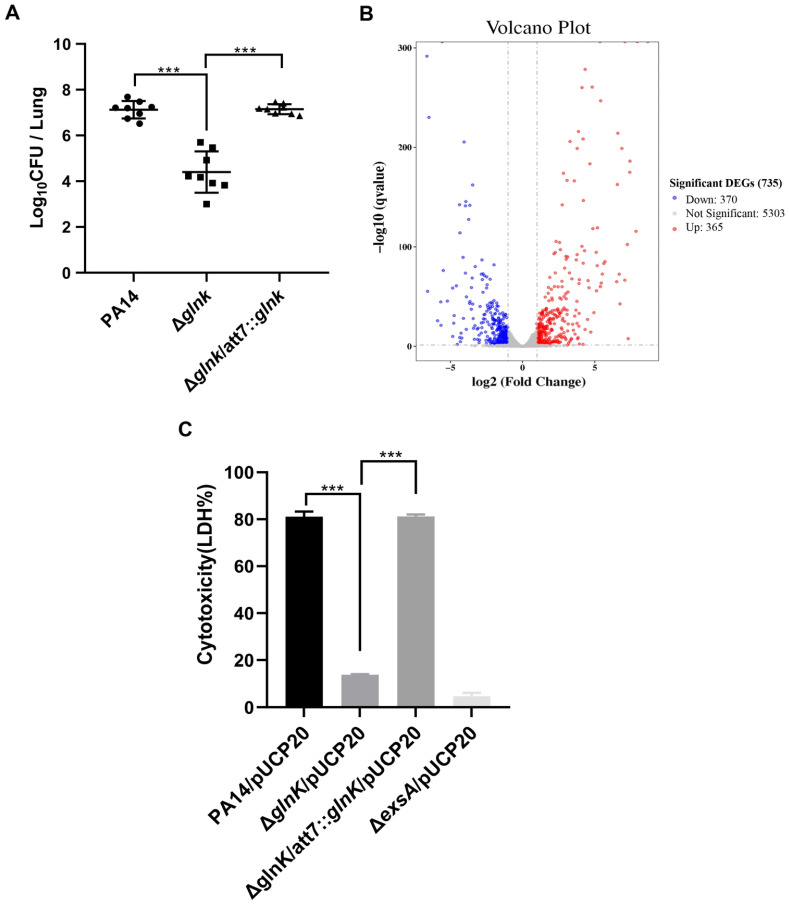
Roles of Glnk in bacterial virulence and gene regulation. (**A**) Bacterial colonization in the murine acute pneumonia model (*n* = 8). Mice were intranasally challenged with 4 × 10^6^ CFU of wild-type PA14, *glnK* mutant (Δ*glnK*), or the complemented strain (*glnK*/att7::*glnK*). Circles, squares and triangles represent individual mice infected with the indicated strains, respectively. At 12 hpi, mice were sacrificed and bacterial loads in the lungs were assessed. *** *p* < 0.001 by ANOVA/Dunnett’s multiple comparison test. (**B**) Volcano plot of differentially expressed genes (DEGs) in PA14 and the Δ*glnK* mutant grown in LB. (**C**) The relative cytotoxicity of Δ*glnK* compared to the PA14 wild-type strain. A549 cells were infected with the indicated strains at an MOI of 50. At 2.5 h post-infection (hpi), the cytotoxicity was determined by the lactate dehydrogenase (LDH) release assay. *** *p* < 0.001 by ANOVA/Dunnett’s multiple comparison test.

The upregulation of *glnK* in mouse lungs and BALF indicated a potential role of GlnK in bacterial virulence. We thus infected mice with a *glnK* transposon insertion mutant (*glnK*::Tn) from the PA14 mutant library. Compared to wild-type PA14, the bacterial loads of the *glnK*::Tn mutant were reduced, which were restored by complementation with a *glnK* gene (Figure S4). To verify the role of GlnK in bacterial virulence, we constructed a *glnK* deletion mutant in wild-type PA14 (Δ*glnK*), which displayed reduced colonization in mouse lungs (Figure 3A). Hematoxylin and eosin (HE) staining revealed alleviated alveolar damage and inflammatory cell infiltration in the lung of the Δ*glnK*-infected mouse (Figure S5). These results demonstrate that GlnK contributes to bacterial virulence during lung infection.

### 3.3. GlnK Is Required for the Expression of the T3SS

**Figure 4:**
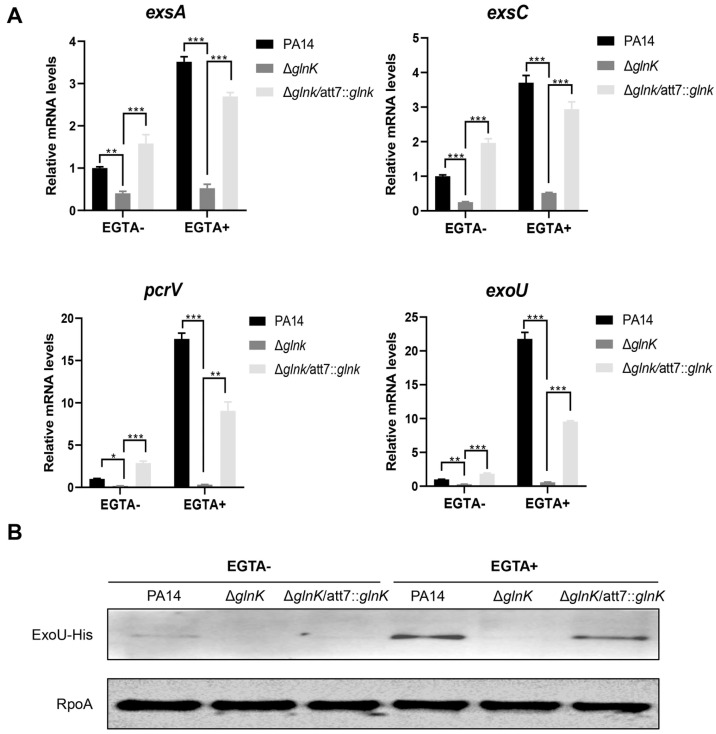
GlnK is required for the expression of the T3SS genes. (**A**) mRNA levels of T3SS genes. The indicated strains were grown to OD_600_ 1.0 in LB with or without EGTA. * *p* < 0.05; ** *p* < 0.01; *** *p* < 0.001 by ANOVA/Dunnett’s multiple comparison test. (**B**) Western blotting of ExoU-His in bacteria carrying P*_exoU_*-*exoU*-His. Bacteria were grown to an OD_600_ of 1.0 in LB containing 150 μg/mL carbenicillin with or without 5 mM EGTA. The RpoA protein was used as the loading control.

**Figure 5:**
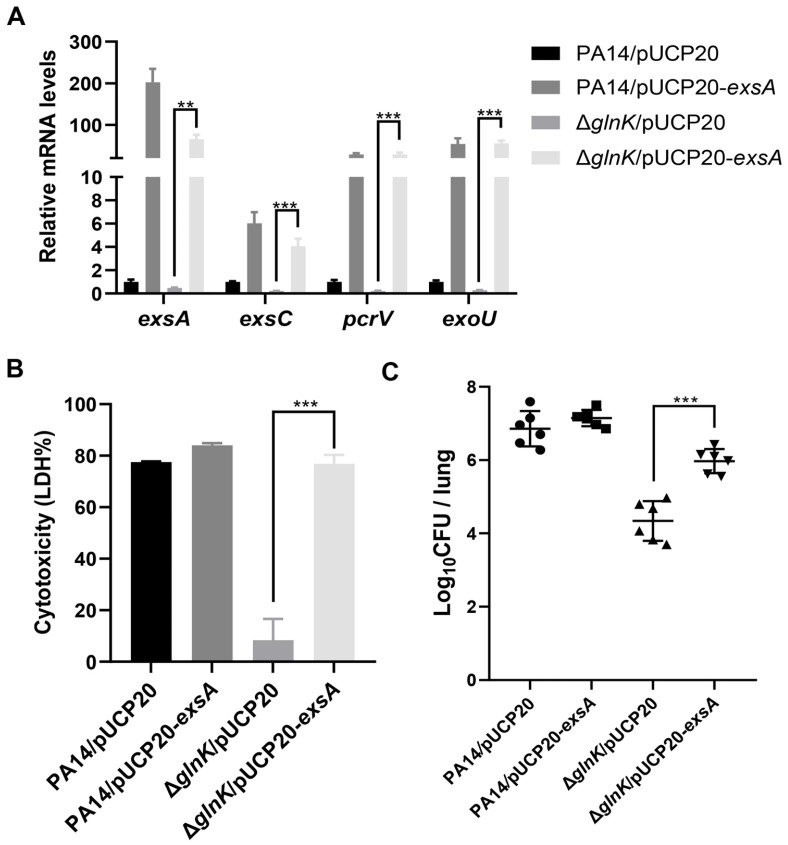
Overexpression of *exsA* restored the T3SS gene expression in the Δ*glnK* mutant. (**A**) T3SS gene transcription analysis. Indicated strains were cultured in LB to an OD_600_ of 1.0. mRNA levels of the T3SS genes were measured by RT-qPCR. (**B**) Cytotoxicity assessment with A549 cells. A549 cells were infected with the indicated strains (MOI = 50). Cytotoxicity was assessed using the lactate dehydrogenase (LDH) release assay. (**C**) Bacterial loads of the indicated strains in the murine acute pneumonia model: circles (PA14/pUCP20), squares (PA14/pUCP20-*exsA*), triangles (Δ*glnK*/pUCP20 and Δ*glnK*/pUCP20-*exsA*).** *p* < 0.01; *** *p* < 0.001 by ANOVA/Dunnett’s multiple comparison test.

To elucidate the mechanism of GlnK-mediated regulation on *P. aeruginosa* virulence, we performed transcriptomic analysis. A total of 735 genes were differentially expressed (>2-fold change) in wild-type PA14 and the Δ*glnK* mutant (Figure 3B). Of note, the T3SS genes were downregulated in the Δ*glnK* mutant (Table 1). RT-qPCR verified downregulation of the T3SS regulatory genes *exsA* and *exsC*, structural gene *pcrV,* and the effector protein gene *exoU* in the Δ*glnK* mutant (Figure 4A). By using a 6 × His-tagged *exoU* driven by its native promoter (P*_exoU_*-*exoU*-His), we further verified the defective expression of ExoU in the Δ*glnK* mutant (Figure 4B). In agreement with the downregulation of T3SS genes, the cytotoxicity was reduced in the Δ*glnK* mutant (Figure 3C). Chromosomal complementation with a *glnK* gene driven by its native promoter restored the expression of the T3SS genes and bacterial cytotoxicity (Figure 3C and Figure 4). To investigate whether GlnK controls bacterial virulence through the T3SS, we overexpressed the T3SS master regulator gene *exsA* in the Δ*glnK* mutant, which increased the expression of the T3SS gene as well as the bacterial cytotoxicity and virulence (Figure 5). Altogether, these results demonstrate a role of GlnK in regulating the T3SS.

### 3.4. GlnK Controls the T3SS by Regulating the NtrB/NtrC Two-Component System Through a Negative Feedback Mechanism

**Figure 6:**
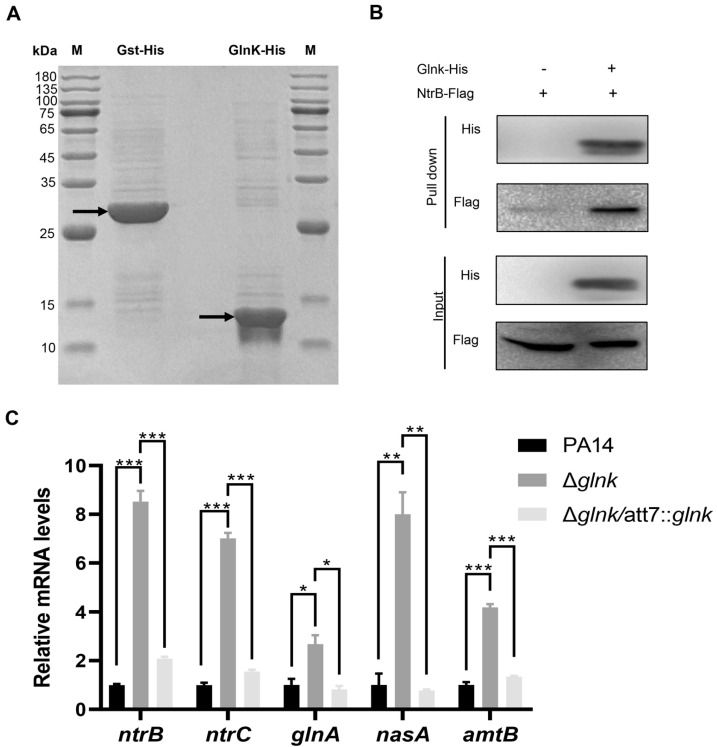
GlnK regulates the activity of the NtrB/NtrC two-component system. (**A**) Identification of GlnK binding proteins. The 6 × His-tagged GlnK (GlnK-His_6_) and GST (Gst-His_6_) were purified using affinity chromatography under a native condition. The purified proteins were analyzed by 15% SDS-PAGE at a constant voltage of 70 V for 30 min, followed by 150 V for 1 h, followed by Coomassie Brilliant Blue staining. M, protein marker. Arrows indicate the purified GlnK-His_6_ and Gst-His_6_. (**B**) Co-purification assay to detect the interaction between GlnK-His_6_ and NtrB-Flag. Cells overexpressing NtrB-Flag (from plasmid pAK1900-*ntrB*-Flag) with the GlnK-His_6_ expression plasmid pMMB67EH-*glnK*-His or the empty vector were grown in LB. The expression of GlnK-His_6_ was induced by 1 mM IPTG for 4 h. Bacterial proteins were subjected to purification with Ni-NTA Agarose Beads (QIAGEN). The eluted protein samples were analyzed by Western blotting with appropriate antibodies. (**C**) PA14, the Δ*glnK* mutant and the complemented strain (Δ*glnK*/att7::*glnK*) were grown in LB. The mRNA levels of *ntrB*, *ntrC*, and nitrogen assimilation genes were determined by RT-qPCR. * *p* < 0.05; ** *p* < 0.01; *** *p* < 0.001 by ANOVA/Dunnett’s multiple comparison test.

Since GlnK exerts its regulatory function by binding to target proteins, we performed affinity chromatography using a C-terminal 6 × His-tagged GlnK (GlnK-His_6_) to decipher its regulatory mechanism on the T3SS. A C-terminal 6 × His-tagged GST (GST-His_6_) was used as a control (Figure 6A). The purified proteins were subjected to mass spectrometry (MS) analyses. Compared to the control group, the known GlnK-interacting proteins, including ArgB, RelA, Rho, TrkA, GlnD, and NtrB were enriched (Table S1) [24]. However, no T3SS-related proteins were identified. A previous study demonstrated that the NtrB-NtrC system is involved in the regulation of bacterial virulence factors [34]. In addition, carbon–nitrogen balance has been shown to regulate the T3SS [35,36]. Given the role of GlnK in maintaining carbon–nitrogen balance through regulating NtrB-NtrC and glutamine synthetase activities, we suspected that GlnK might regulate the T3SS by modulating the NtrB-NtrC system.

**Figure 7:**
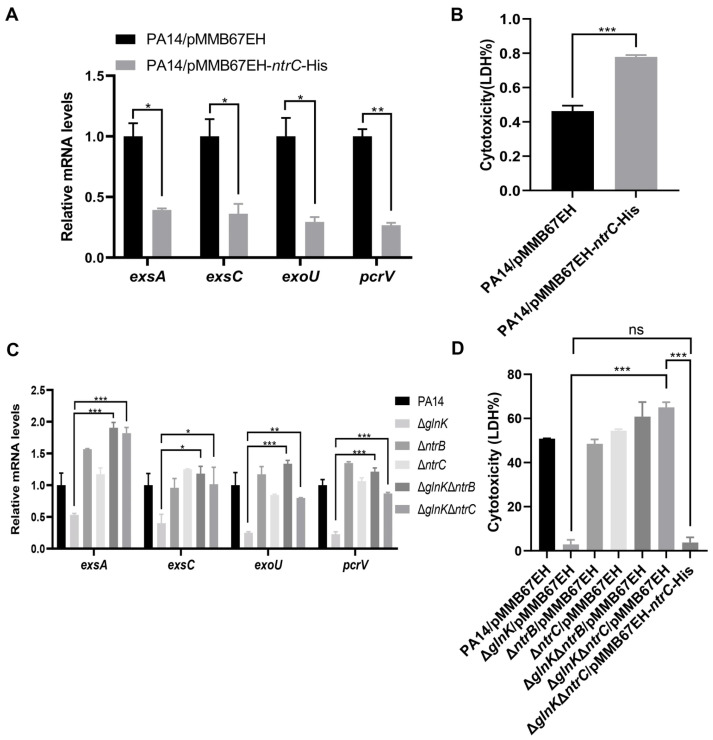
GlnK regulates the T3SS through the NtrB/NtrC two-component system. (**A**) mRNA levels of T3SS genes in PA14 and PA14 overexpressing *ntrC*. * *p* < 0.05; ** *p *< 0.01 by Student’s *t*-test. (**B**) Lactate dehydrogenase (LDH) release assay with PA14 and PA14 overexpressing *ntrC*. A549 cells were infected with the indicated strains at an MOI of 50, and 1 mM isopropyl β-D-1-thiogalactopyranoside (IPTG) was added into the culture medium. At 4.5 hpi, the cytotoxicity was determined. *** *p* < 0.001 by Student’s *t*-test. (**C**) Expression levels of the T3SS genes. The mRNA levels were examined by RT-qPCR. * *p* < 0.05; ** *p* < 0.01; *** *p* < 0.001 by ANOVA/Dunnett’s multiple comparison test. (**D**) Cytotoxicity assay of the indicated strains. A549 cells were infected with the indicated strains (MOI = 50) in IPTG-supplemented medium (1 mM), and cytotoxicity was determined by LDH release assay at 4.5 hpi. ns, not significant; *** *p* < 0.001 by ANOVA/Dunnett’s multiple comparison test.

To verify this hypothesis, we first confirmed the interaction between GlnK and NtrB by affinity chromatography (Figure 6B). Then we found that mutation of *glnK* resulted in upregulation of *ntrB*, *ntrC*, and nitrogen assimilation genes (Figure 6C), indicating a negative regulation of the NtrB-NtrC system by GlnK. Overexpression of *ntrC* reduced the expression of the T3SS genes and bacterial cytotoxicity (Figure 7A,B), demonstrating a negative regulatory role of NtrC on the T3SS. Deletion of *ntrB* or *ntrC* in the Δ*glnK* mutant restored the expression levels of *exsA*, *exsC*, *exoU*, and *pcrV*, as well as bacterial cytotoxicity (Figure 7C,D). Overexpression of *ntrC* in the Δ*glnK*Δ*ntrC* double mutant reduced the bacterial cytotoxicity to a similar level to the Δ*glnK* mutant (Figure 7D). Collectively, these results reveal a role of GlnK in regulating the T3SS by modulating the NtrB-NtrC system.

## 4. Discussion

In this study, we demonstrated that GlnK modulates the T3SS by regulating the NtrB-NtrC system through a negative feedback mechanism. During colonization in mouse lung or growth in BALF, the NtrB-NtrC system is activated. Supplementation of NH_4_Cl in the BALF reduced the expression of *ntrB*, *ntrC*, and nitrogen assimilation genes, indicating a nitrogen-limiting environment in vivo. Unlike most typical two-component regulatory systems, the sensor NtrB is an intracellular protein [37]. It is a bifunctional enzyme with both kinase and phosphatase activities which is regulated by GlnK. Under nitrogen-replete conditions, unmodified GlnK binds to NtrB, favoring its phosphatase activity, thus leading to dephosphorylation of NtrC. Conversely, under nitrogen-limitation conditions, GlnK undergoes uridylylation to form GlnK~UMP. GlnK~UMP loses its ability to bind NtrB. As a result, NtrB switches to its kinase activity, driving increased phosphorylation of NtrC (NtrC~P) [38,39].

Besides the nitrogen acquisition genes, NtrC~P upregulates the *glnK* gene, thereby forming a negative feedback loop. Specially, deletion of *glnK* might keep NtrB in its kinase-active state and subsequently promote the formation of NtrC~P, which traps the bacteria in a nitrogen starvation response state. It has been demonstrated that bacteria may prioritize survival processes over energy-costly virulence traits in response to nutrient starvation. For instance, culture of *Salmonella typhimurium* in minimal media reduced its ability to invade epithelial cells [40].

As for *P. aeruginosa*, growth in mannitol resulted in lower T3SS gene expression than those grown in the preferred carbon source succinate, which also suggests a role of catabolite repression in regulating the T3SS [41]. Indeed, the catabolite repressor Crc is required for the expression of T3SS genes [35]. Crc in partnership with the RNA chaperone Hfq controls gene expression at the post-transcriptional level [42,43]. The activity of Crc is antagonized by a small RNA CrcZ, whose expression is regulated by the CbrA-CbrB two-component system in response to different carbon sources [44]. Besides carbon sources, the CbrA-CbrB two-component system might also be involved in responding to carbon–nitrogen imbalance [45]. A previous study demonstrated that perturbation of histidine uptake and catabolism lead to defective T3SS, likely due to carbon–nitrogen imbalance [36]. Given the role of GlnK in maintaining carbon–nitrogen balance, further studies are warranted to investigate the interconnection between the NtrB-NtrC and CbrA-CbrB two-component systems and their roles in regulating the T3SS.

Since our data revealed activation of the NtrB-NtrC two-component system in the *glnK* mutant, it is possible that NtrB-NtrC upregulates a negative regulator of the T3SS. The regulator might repress the transcription of the T3SS genes and the activity of ExsA or other T3SS positive regulators. Another possibility is that NtrB-NtrC upregulates a small RNA that represses the T3SS. Currently, efforts are being made to elucidate the regulatory pathway.

Collectively, the GlnK-NtrB-NtrC-T3SS regulatory axis identified in this study adds critical depth to our understanding of how *P. aeruginosa* integrates metabolic cues with virulence.

## 5. Conclusions

In this work, we demonstrate that GlnK, a central regulator of nitrogen metabolism, functions as a critical link between nutrient sensing and virulence regulation. Our results reveal a GlnK–NtrB–NtrC–T3SS regulatory axis that allows *P. aeruginosa* to adapt to the pulmonary nutrient condition and fine-tune the expression of the virulence factor type III secretion system. These findings bridge metabolic adaptation and pathogenicity by elucidating a “nitrogen-sensing–virulence” signaling pathway that is essential for bacterial pathogenesis. This work sheds light on the coordination of bacterial metabolism and virulence during infection.

## Figures and Tables

**Table 1:** Downregulated T3SS genes in the Δ*glnK* mutant.

Gene ID	Gene Name	Description	log_2_FC ^a^
PA14_42250	*pscL*	T3SS stator protein PscL	−1.18
PA14_42260	*pscK*	T3SS sorting platform protein PscK	−1.35
PA14_42270	*pscJ*	T3SS inner membrane ring lipoprotein PscJ	−1.06
PA14_42280	*pscI*	T3SS inner rod subunit PscI	−0.91
PA14_42290	*pscH*	T3SS polymerization control protein PscH	−1.53
PA14_42300	*pscG*	T3SS chaperone PscG	−1.68
PA14_42310	*pscF*	T3SS needle filament protein PscF	−1.15
PA14_42320	*pscE*	T3SS co-chaperone PscE	−2.54
PA14_42340	*pscD*	T3SS inner membrane ring subunit PscD	−1.01
PA14_42350	*pscC*	T3SS outer membrane ring subunit PscC	−1.18
PA14_42360	*pscB*	T3SS chaperone PscB	−1.51
PA14_42380	*exsD*	T3SS regulon anti-activator ExsD	−1.18
PA14_42390	*exsA*	T3SS regulon transcriptional activator ExsA	−1.17
PA14_42400	*exsB*	T3SS pilotin ExsB	−1.79
PA14_42410	*exsE*	T3SS regulon translocated regulator ExsE	−2.12
PA14_42430	*exsC*	T3SS regulatory chaperone ExsC	−1.53
PA14_42440	*popD*	T3SS translocon subunit PopD	−2.27
PA14_42450	*popB*	T3SS translocon subunit PopB	−2.49
PA14_42460	*pcrH*	T3SS chaperone PcrH	−3.08
PA14_42470	*pcrV*	T3SS needle tip protein PcrV	−2.06
PA14_42480	*pcrG*	T3SS chaperone PcrG	−3.12
PA14_42490	*pcrR*	T3SS chaperone PcrR	−1.41
PA14_42500	*pcrD*	T3SS export apparatus subunit PcrD	−0.68
PA14_42510	*pcr4*	T3SS chaperone	−2.09
PA14_42530	*pcr2*	T3SS protein	−1.68
PA14_42540	*pcr1*	T3SS gatekeeper subunit Pcr1	−1.36
PA14_42550	*popN*	T3SS gatekeeper subunit PopN	−1.64
PA14_42570	*pscN*	T3SS ATPase PscN	−1.34
PA14_42580	*pscO*	T3SS central stalk protein PscO	−1.48
PA14_42600	*pscP*	T3SS needle length determinant PscP	−0.80
PA14_42610	*pscQ*	T3SS cytoplasmic ring protein PscQ	−0.67
PA14_42620	*pscR*	T3SS export apparatus subunit PscR	−0.80
PA14_42630	*pscS*	T3SS export apparatus subunit PscS	−3.32
PA14_42640	*pscT*	T3SS export apparatus subunit PscT	−0.78
PA14_42660	*pscU*	T3SS export apparatus subunit PscU	−1.03
PA14_51530	*exoU*	T3SS effector cytotoxin ExoU	−1.19
PA14_00560	*exoT*	T3SS effector bifunctional cytotoxin exoenzyme T	−1.77

^a^ FC, fold change (Δ*glnK* vs. wild-type PA14).

## Data Availability

Raw RNA-seq data generated in this study have been deposited in the National Center for Biotechnology Information (NCBI) Sequence Read Archive (SRA) at https://www.ncbi.nlm.nih.gov/bioproject, under BioProject number: PRJNA1347373, accessed on 21 October 2025.

## Supplementary Materials

The following supporting information can be downloaded at: https://www.mdpi.com/article/10.3390/microorganisms14020339/s1, Figure S1: The expression of *glnK* in wild-type PA14 in response to the mouse lung environment; Figure S2: Sequence analysis of PA14 NtrC and *glnK* promoter; Figure S3: Electrophoretic mobility shift assay (EMSA) for negative controls of NtrC binding to the *glnK* promoter region; Figure S4: Bacterial colonization in the murine acute pneumonia model; Figure S5: Representative H&E staining of lung tissues from mice infected with the indicated strains. Table S1: Mass spectrometry data of GlnK-interacting proteins; Table S2: Primers used in this study; Table S3: Plasmids and strains used in this study.

## Abbreviations

The following abbreviations are used in this manuscript:

T3SS	Type III secretion system
MOI	Multiplicity of infection

## References

[1] Jault P., Leclerc T., Jennes S., Pirnay J.P., Que Y.A., Resch G., Rousseau A.F., Ravat F., Carsin H., Le Floch R. (2019). Efficacy and tolerability of a cocktail of bacteriophages to treat burn wounds infected by *Pseudomonas aeruginosa* (PhagoBurn): A randomised, controlled, double-blind phase 1/2 trial. Lancet Infect. Dis..

[2] Pouget C., Dunyach-Remy C., Magnan C., Pantel A., Sotto A., Lavigne J.P. (2022). Polymicrobial Biofilm Organization of *Staphylococcus aureus* and *Pseudomonas aeruginosa* in a Chronic Wound Environment. Int. J. Mol. Sci..

[3] Weiner I., Kahan-Hanum M., Buchstab N., Zelcbuch L., Navok S., Sherman I., Nicenboim J., Axelrod T., Berko-Ashur D., Olshina M. (2025). Phage therapy with nebulized cocktail BX004-A for chronic *Pseudomonas aeruginosa* infections in cystic fibrosis: A randomized first-in-human trial. Nat. Commun..

[4] Scribani Rossi C., Barrientos-Moreno L., Paone A., Cutruzzola F., Paiardini A., Espinosa-Urgel M., Rinaldo S. (2022). Nutrient Sensing and Biofilm Modulation: The Example of L-arginine in Pseudomonas. Int. J. Mol. Sci..

[5] Zhou M., Huang Y., Zhang Y., Wang Q., Ma Y., Shao S. (2022). Roles of virulence regulator ToxR in viable but non-culturable formation by controlling reactive oxygen species resistance in pathogen *Vibrio alginolyticus*. Microbiol. Res..

[6] Matz C., Moreno A.M., Alhede M., Manefield M., Hauser A.R., Givskov M., Kjelleberg S. (2008). *Pseudomonas aeruginosa* uses type III secretion system to kill biofilm-associated amoebae. ISME J..

[7] Reuven A.D., Katzenell S., Mwaura B.W., Bliska J.B. (2025). ExoS effector in *Pseudomonas aeruginosa* Hyperactive Type III secretion system mutant promotes enhanced Plasma Membrane Rupture in Neutrophils. PLoS Pathog..

[8] Brutinel E.D., King J.M., Marsden A.E., Yahr T.L. (2012). The distal ExsA-binding site in *Pseudomonas aeruginosa* type III secretion system promoters is the primary determinant for promoter-specific properties. J. Bacteriol..

[9] Williams McMackin E.A., Djapgne L., Corley J.M., Yahr T.L. (2019). Fitting Pieces into the Puzzle of *Pseudomonas aeruginosa* Type III Secretion System Gene Expression. J. Bacteriol..

[10] Chen H., Gong X., Fan Z., Xia Y., Jin Y., Bai F., Cheng Z., Pan X., Wu W. (2023). *Pseudomonas aeruginosa* Citrate Synthase GltA Influences Antibiotic Tolerance and the Type III Secretion System through the Stringent Response. Microbiol. Spectr..

[11] Dasgupta N., Ashare A., Hunninghake G.W., Yahr T.L. (2006). Transcriptional induction of the *Pseudomonas aeruginosa* type III secretion system by low Ca2+ and host cell contact proceeds through two distinct signaling pathways. Infect. Immun..

[12] Urbanowski M.L., Lykken G.L., Yahr T.L. (2005). A secreted regulatory protein couples transcription to the secretory activity of the *Pseudomonas aeruginosa* type III secretion system. Proc. Natl. Acad. Sci. USA.

[13] Vakulskas C.A., Brady K.M., Yahr T.L. (2009). Mechanism of transcriptional activation by *Pseudomonas aeruginosa* ExsA. J. Bacteriol..

[14] Arif S.J., Hoffman K.M., Flynn J.M., Wiggen T.D., Lucas S.K., Villarreal A.R., Gilbertsen A.J., Dunitz J.M., Hunter R.C. (2025). Host- and microbial-mediated mucin degradation differentially shape *Pseudomonas aeruginosa* physiology and gene expression. PLoS Pathog..

[15] Pan X., Fan Z., Chen L., Liu C., Bai F., Wei Y., Tian Z., Dong Y., Shi J., Chen H. (2020). PvrA is a novel regulator that contributes to *Pseudomonas aeruginosa* pathogenesis by controlling bacterial utilization of long chain fatty acids. Nucleic Acids Res..

[16] Pan X., Liang H., Zhao X., Zhang Q., Chen L., Yue Z., Yin L., Jin Y., Bai F., Cheng Z. (2023). Regulatory and structural mechanisms of PvrA-mediated regulation of the PQS quorum-sensing system and PHA biosynthesis in *Pseudomonas aeruginosa*. Nucleic Acids Res..

[17] Kuypers M.M.M., Marchant H.K., Kartal B. (2018). The microbial nitrogen-cycling network. Nat. Rev. Microbiol..

[18] van Heeswijk W.C., Westerhoff H.V., Boogerd F.C. (2013). Nitrogen assimilation in *Escherichia coli*: Putting molecular data into a systems perspective. Microbiol. Mol. Biol. Rev..

[19] Adler S.P., Purich D., Stadtman E.R. (1975). Cascade control of *Escherichia coli* glutamine synthetase. Properties of the PII regulatory protein and the uridylyltransferase-uridylyl-removing enzyme. J. Biol. Chem..

[20] Bolay P., Rozbeh R., Muro-Pastor M.I., Timm S., Hagemann M., Florencio F.J., Forchhammer K., Klahn S. (2021). The Novel P(II)-Interacting Protein PirA Controls Flux into the Cyanobacterial Ornithine-Ammonia Cycle. mBio.

[21] Gosztolai A., Schumacher J., Behrends V., Bundy J.G., Heydenreich F., Bennett M.H., Buck M., Barahona M. (2017). GlnK Facilitates the Dynamic Regulation of Bacterial Nitrogen Assimilation. Biophys. J..

[22] Javelle A., Severi E., Thornton J., Merrick M. (2004). Ammonium sensing in *Escherichia coli*. Role of the ammonium transporter AmtB and AmtB-GlnK complex formation. J. Biol. Chem..

[23] Pedro-Roig L., Lange C., Bonete M.J., Soppa J., Maupin-Furlow J. (2013). Nitrogen regulation of protein-protein interactions and transcript levels of GlnK PII regulator and AmtB ammonium transporter homologs in Archaea. MicrobiologyOpen.

[24] Gerhardt E.C.M., Parize E., Gravina F., Pontes F.L.D., Santos A.R.S., Araujo G.A.T., Goedert A.C., Urbanski A.H., Steffens M.B.R., Chubatsu L.S. (2020). The Protein-Protein Interaction Network Reveals a Novel Role of the Signal Transduction Protein PII in the Control of c-di-GMP Homeostasis in *Azospirillum brasilense*. mSystems.

[25] Xu M., Tang M., Chen J., Yang T., Zhang X., Shao M., Xu Z., Rao Z. (2020). PII Signal Transduction Protein GlnK Alleviates Feedback Inhibition of N-Acetyl-l-Glutamate Kinase by l-Arginine in Corynebacterium glutamicum. Appl. Environ. Microbiol..

[26] Pokorzynski N.A.-O., Groisman E.A.-O. (2023). How Bacterial Pathogens Coordinate Appetite with Virulence. Microbiol. Mol. Biol. Rev..

[27] Choi K.H., Schweizer H.P. (2006). Mini-Tn7 insertion in bacteria with single attTn7 sites: Example *Pseudomonas aeruginosa*. Nat. Protoc..

[28] Kuang Z., Bennett R.C., Lin J.A.-O., Hao Y., Zhu L.A.-O., Akinbi H.T., Lau G.W. (2020). Surfactant phospholipids act as molecular switches for premature induction of quorum sensing-dependent virulence in *Pseudomonas aeruginosa*. Virulence.

[29] Li S., Weng Y., Li X., Yue Z., Chai Z., Zhang X., Gong X., Pan X.A.-O., Jin Y., Bai F. (2021). Acetylation of the CspA family protein CspC controls the type III secretion system through translational regulation of exsA in *Pseudomonas aeruginosa*. Nucleic Acids Res..

[30] Qu J., Yin L., Qin S., Sun X., Gong X., Li S., Pan X., Jin Y., Cheng Z., Jin S. (2025). Identification of the *Pseudomonas aeruginosa* AgtR-CspC-RsaL pathway that controls Las quorum sensing in response to metabolic perturbation and *Staphylococcus aureus*. PLoS Pathog..

[31] Huergo L.F., Chubatsu L.S., Souza E.M., Pedrosa F.O., Steffens M.B.R., Merrick M. (2006). Interactions between PII proteins and the nitrogenase regulatory enzymes DraT and DraG in *Azospirillum brasilense*. FEBS Lett..

[32] Hervas A.B., Canosa I., Little R., Dixon R., Santero E. (2009). NtrC-dependent regulatory network for nitrogen assimilation in *Pseudomonas putida*. J. Bacteriol..

[33] Schumacher J., Behrends V., Pan Z., Brown D.R., Heydenreich F., Lewis M.R., Bennett M.H., Razzaghi B., Komorowski M., Barahona M. (2013). Nitrogen and carbon status are integrated at the transcriptional level by the nitrogen regulator NtrC in vivo. mBio.

[34] Alford M.A., Baquir B., An A., Choi K.G., Hancock R.E.W. (2021). NtrBC Selectively Regulates Host-Pathogen Interactions, Virulence, and Ciprofloxacin Susceptibility of *Pseudomonas aeruginosa*. Front. Cell Infect. Microbiol..

[35] Gil-Gil T., Cuesta T., Hernando-Amado S., Reales-Calderon J.A., Corona F., Linares J.F., Martinez J.L. (2023). Virulence and Metabolism Crosstalk: Impaired Activity of the Type Three Secretion System (T3SS) in a *Pseudomonas aeruginosa* Crc-Defective Mutant. Int. J. Mol. Sci..

[36] Rietsch A., Wolfgang M.C., Mekalanos J.J. (2004). Effect of metabolic imbalance on expression of type III secretion genes in *Pseudomonas aeruginosa*. Infect. Immun..

[37] Song Y., Peisach D., Pioszak A.A., Xu Z., Ninfa A.J. (2004). Crystal structure of the C-terminal domain of the two-component system transmitter protein nitrogen regulator II (NRII.; NtrB), regulator of nitrogen assimilation in *Escherichia coli*. Biochemistry.

[38] Atkinson M.R., Kamberov E.S., Weiss R.L., Ninfa A.J. (1994). Reversible uridylylation of the *Escherichia coli* PII signal transduction protein regulates its ability to stimulate the dephosphorylation of the transcription factor nitrogen regulator I (NRI or NtrC). J. Biol. Chem..

[39] Atkinson M.R., Ninfa A.J. (1999). Characterization of the GlnK protein of *Escherichia coli*. Mol. Microbiol..

[40] Yurist-Doutsch S., Arrieta M.C., Tupin A., Valdez Y., Antunes L.C., Yen R., Finlay B.B. (2016). Nutrient Deprivation Affects Salmonella Invasion and Its Interaction with the Gastrointestinal Microbiota. PLoS ONE.

[41] Dong Y.H., Zhang X.F., Zhang L.H. (2013). The global regulator Crc plays a multifaceted role in modulation of type III secretion system in *Pseudomonas aeruginosa*. MicrobiologyOpen.

[42] Malecka E.M., Bassani F., Dendooven T., Sonnleitner E., Rozner M., Albanese T.G., Resch A., Luisi B., Woodson S., Blasi U. (2021). Stabilization of Hfq-mediated translational repression by the co-repressor Crc in *Pseudomonas aeruginosa*. Nucleic Acids Res..

[43] Sonnleitner E., Wulf A., Campagne S., Pei X.Y., Wolfinger M.T., Forlani G., Prindl K., Abdou L., Resch A., Allain F.H. (2018). Interplay between the catabolite repression control protein Crc, Hfq and RNA in Hfq-dependent translational regulation in *Pseudomonas aeruginosa*. Nucleic Acids Res..

[44] Sonnleitner E., Abdou L., Haas D. (2009). Small RNA as global regulator of carbon catabolite repression in *Pseudomonas aeruginosa*. Proc. Natl. Acad. Sci. USA.

[45] Nishijyo T., Haas D., Itoh Y. (2001). The CbrA-CbrB two-component regulatory system controls the utilization of multiple carbon and nitrogen sources in *Pseudomonas aeruginosa*. Mol. Microbiol..

