# Correction: Sun et al. GlnK Regulates the Type III Secretion System by Modulating NtrB-NtrC Homeostasis in *Pseudomonas aeruginosa*. *Microorganisms* 2026, *14*, 339

**DOI:** 10.3390/microorganisms14071485

**Published:** 2026-07-07

**Authors:** Xiaomeng Sun, Qitong Du, Yiming Li, Xuetao Gong, Yu Zhang, Yongxin Jin, Shouguang Jin, Weihui Wu

**Affiliations:** State Key Laboratory of Medicinal Chemical Biology, Key Laboratory of Molecular Microbiology and Technology of the Ministry of Education, Department of Microbiology, College of Life Sciences, Nankai University, Tianjin 300071, China; 1120220629@mail.nankai.edu.cn (X.S.);

## Error in Figure

In the original publication [1], there was a mistake in Figure 7B as published. Data values were calculated by $\left[\frac{A_{infected}-A_{blank}}{A_{control}-A_{blank}}\right]$, but the proper formula for cytotoxicity percentage is $Cytotoxicity\;(\%)=\left[1-\frac{A_{infected}-A_{blank}}{A_{control}-A_{blank}}\right]\times 100$. The corrected Figure 7 appears as below.

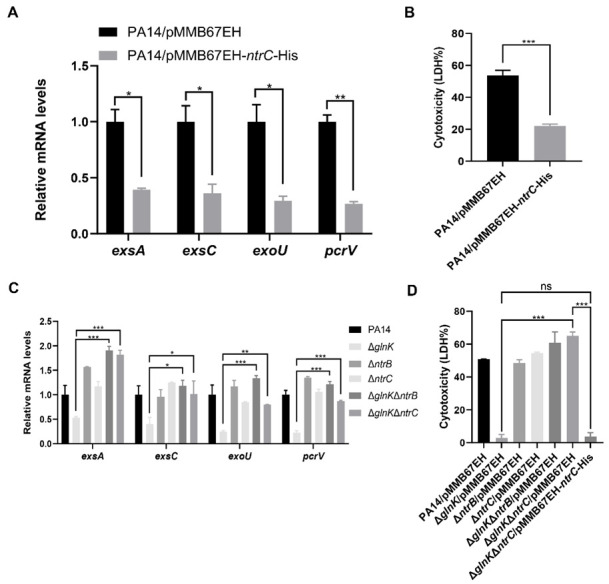



## Text Correction

There was an error in the original publication [1]. The cytotoxicity assay method is not clear enough.

A correction has been made to Section 2.8:

*2.8. Cytotoxicity Assay*

Lactate dehydrogenase (LDH) assays were used to quantify intracellular LDH in adherent viable cells for cytotoxicity assessment using an LDH Cytotoxicity Assay Kit (Solarbio, Tongzhou, China) following the manufacturer’s instructions. A549 cells were seeded into 24-well plates (2 × 10^5^ cells per well) and cultured in RPMI 1640 medium supplemented with 10% fetal bovine serum (FBS) at 37 °C with 5% CO_2_. Indicated bacterial strains were cultured overnight in LB. On the following day, the bacteria were subcultured into fresh LB and grown to log phase (OD_600_ = 1.0), followed by washing once and resuspension in RPMI 1640 medium. A549 cells were infected with bacteria at a multiplicity of infection (MOI) of 50, followed by centrifugation at 500× *g* for 5 min to synchronize bacterial cell contact. The addition of RPMI 1640 medium to parallel wells was used as a blank control. For bacterial strains requiring inducible expression, 1 mM isopropyl β-D-1-thiogalactopyranoside (IPTG) was added to the culture. At 2.5 or 4.5 hours post-infection, the culture medium was removed, and adherent live cells were lysed with the kit-supplied LDH release buffer. After addition of the reaction buffer, absorbance at 490 nm was measured with a multimode microplate reader (Thermo Fisher Scientific, Waltham, MA, USA). The cytotoxicity rate was calculated with the following formula:

$$Cytotoxicity\;(\%)=\left[1-\frac{A_{infected}-A_{blank}}{A_{control}-A_{blank}}\right]\times 100$$

A_infected_ and A_control_ denote absorbance of intracellular LDH from adherent viable cells with and without bacterial infection, respectively, and A_blank_ denotes absorbance of the blank.

The authors state that the scientific conclusions are unaffected. This correction was approved by the Academic Editor. The original publication has also been updated.
