# Supplementary material for: GlnK Regulates the Type III Secretion System by Modulating NtrB-NtrC Homeostasis in Pseudomonas aeruginosa
Source: Microorganisms. 2026 Feb 2;14(2):339. doi: 10.3390/microorganisms14020339 (PMC12942738; doi:10.3390/microorganisms14020339)
Supplement: Supplementary file 1 [file microorganisms-14-00339-s001.zip › Supplementary figures and tables.pdf]

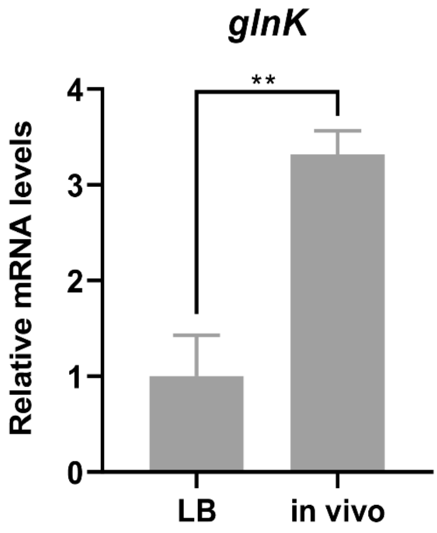

Supplemental Figure S1. The expression of *glnK* in wild-type PA14 in response to the mouse lung environment. mRNA levels of *glnK* in PA14 cells isolated from mouse lungs at 6 hours post-infection and those cultured in LB (OD_{600}=1.0). **, $p < 0.01$ by Student's *t*-test.

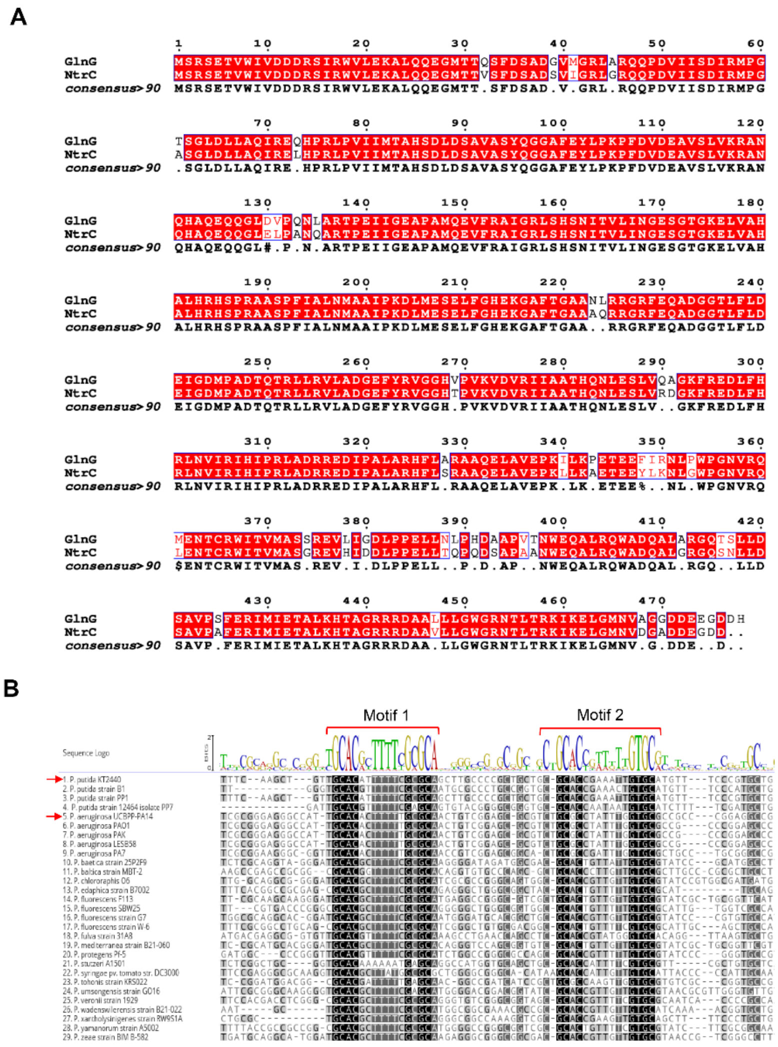

Supplemental Figure S2. Sequence analysis of PA14 NtrC and *glnK* promoter. (A) Amino acid sequence alignment of NtrC from *P. aeruginosa* PA14 (NtrC) and *P. putida* KT2440 (GlnG) using Clustal Omega, followed by visualization via ESPript 3.0. Red-shaded regions indicate amino acid residues that are conserved across the aligned sequences. (B) Comparison of NtrC binding sites in the *glnK* promoters of *Pseudomonads*. DNA sequences were derived from 29 *Pseudomonas* strains with putative NtrC binding sites in the *glnK* promoter region. The *glnK* promoters of *Pseudomonas putida* KT2440 and *P. aeruginosa* PA14 are indicated by arrows, with the predicted NtrC-binding core motifs (Motif 1 and Motif 2) are marked. Sequence alignment and logo were generated using the Geneious Prime (https://www.geneious.com).

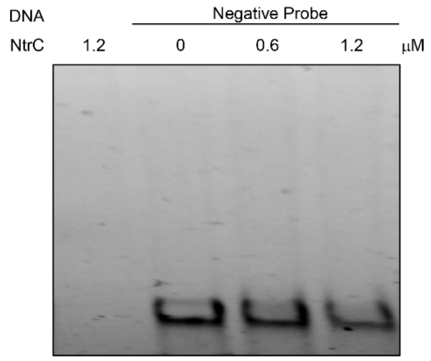

Supplemental Figure S3. Electrophoretic mobility shift assay (EMSA) for negative controls of NtrC binding to the *glnK* promoter region. NtrC fails to bind to a DNA probe generated by deleting the predicted NtrC-binding motifs (Motif 1 and Motif 2), while maintaining the same length as the target *glnK* promoter probe.

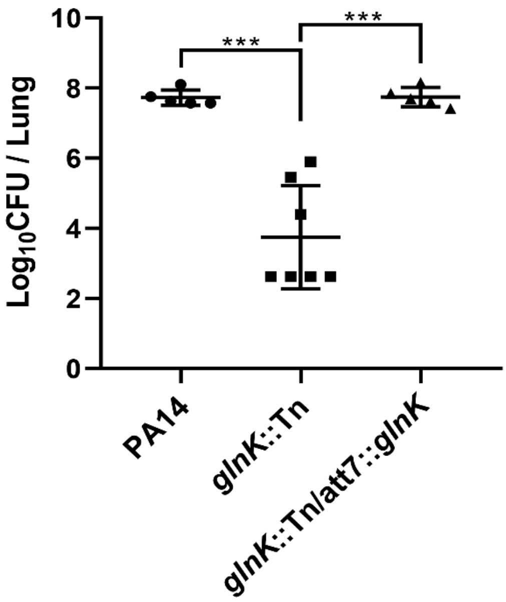

Supplemental Figure S4. Bacterial colonization in the murine acute pneumonia model. Each mouse was infected intranasally with 4×10^6 CFU of wild-type PA14, a *glnK* transposon mutant (*glnK*::Tn) and the complemented strain (*glnK*::Tn/att7::*glnK*). At 12 hpi, bacterial loads in the lungs were determined by plating. Symbols represent individual mice infected with the indicated strains. Bars represent medians, and error bars represent standard deviations. ***, $P < 0.001$ by ANOVA/ Dunnett's multiple comparison test.

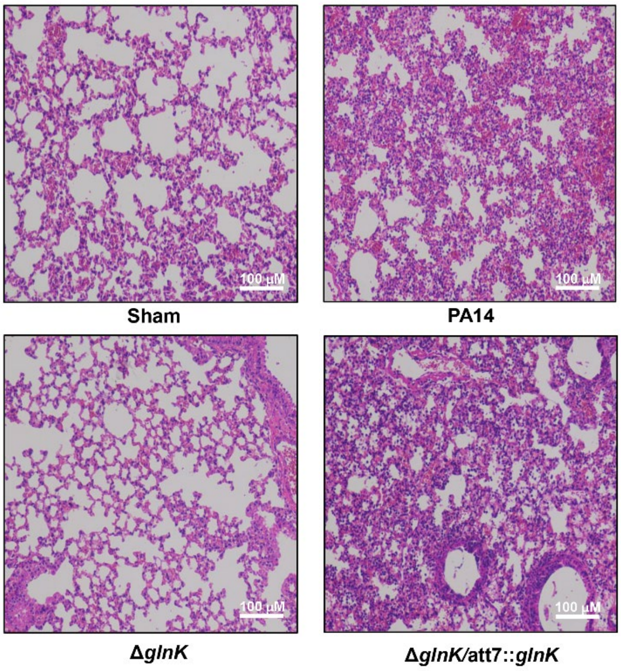

Supplemental Figure S5. Representative H&E staining of lung tissues from mice infected with the indicated strains. Sterilized NaCl 0.9% was used as a sham control. Scale bar = 100 μm.

Table S2. Primers used in this study.

Primer	Sequence (5′→3′)	Purpose
RT-*glnK*-F	ACTGCCATCATCAAGCCG	RT-PCR
RT-*glnK*-R	GCCGAAACCCTTGACCTC	RT-PCR
RT-*ntrB*-F	GACTACACCAACGTCATC	RT-PCR
RT-*ntrB*-R	CCAGCACTTCATGGATGTTG	RT-PCR
RT-*ntrC*-F	GCTGATCAACGGCGAATC	RT-PCR
RT-*ntrC*-R	CCATCAGGTCCTTGGGGATC	RT-PCR
RT-*nasA*-F	GACCCTGTTCGCGGCGTT	RT-PCR
RT-*nasA*-R	CATCATCGCGCGCTGTTG	RT-PCR
RT-*glnA*-F	GTTCTTCGAAGCCGGCAA	RT-PCR
RT-*glnA*-R	GCTGTCGTCCGGCATCAG	RT-PCR
RT-*amtB*-F	GTGCTGTCGATCATGATG	RT-PCR
RT-*amtB*-R	TCGCTGTTGTCGAAATCG	RT-PCR
RT-*exsA*-F	GCTATGTCGTAAGTACCA	RT-PCR
RT-*exsA*-R	GAAGCCTTGTAGAAACTG	RT-PCR
RT-*exsC*-F	CAGCTTCAACCGCCATTG	RT-PCR
RT-*exsC*-R	CGCATACAACTGGACCTTG	RT-PCR
RT-*pcrV*-F	CACGCTCTATGGCTATGC	RT-PCR
RT-*pcrV*-R	AAGGTATCCAGATTGCTCAG	RT-PCR
RT-*exoU*-F	AACACATTAGCAGCGAGAT	RT-PCR
RT-*exoU*-R	AGCAGCAACTCAGAGAAG	RT-PCR
ntrB-LF	CGAGCTCAGTTGCAACTGGTGCTGGACGGCAAACCCTACGGCACGCCC	*ntrB* deletion
ntrB-LR	CTCTGATCGGCTCATGGGGCGGGCAGCTGTTCCAAGGTTGGGCAGG	*ntrB* deletion
ntrB-RF	GGAACAGCTGCCCGCCCCATGAGCCGATCAGAGACCGTCTGGATCGTC	*ntrB* deletion
ntrB-RR	CCAAGCTTGCTCCTGGGCGGCGCGGCTGAGGAAGTGCCGGGCCAG	*ntrB* deletion
ntrC-LF	CGGGATCCTGGAGTACATGAACCCGGCAG	*ntrC* deletion
ntrC-LR	GTGGATCAACGGGTCAATGCACTCCTTGTTCCAGGGGCA	*ntrC* deletion
ntrC-RF	GTGCATTGACCGAATACCTGCCCAAGCCGTTCGAC	*ntrC* deletion
ntrC-RR	CCAAGCTTCGAGCTGGTGATGAATGCCTCTGGAG	*ntrC* deletion
glnK-LF	CGAGCTCGAGTGCGATGGCCAGGT	*glnK* deletion
glnK-LR	TCGGTTGGGCGAAACTCTCTCCCGTGTT	*glnK* deletion
glnK-RF	GAGAGAGTTTCGCCCAACCGAACCCCCAAA	*glnK* deletion
glnK-RR	CGGGATCCCCATCGGACCGGCGGTG	*glnK* deletion
P_{glnK}-F	CGGAATTCCGGCGAACAGGCGGCTG	P_{glnK}-lacZ cloning
P_{glnK}-R	CGGGATCCGAAACTCTCTCCCGTGTTTGG	P_{glnK}-lacZ cloning
Aneal-P_{glnK}-F	CCATTGCACACTTTTTGCGCAACTGTCGGAGCGCGTCTGCGCCTATTTGGTGCGCCGCC	EMSA
Aneal-P_{glnK}-R	GGCGGCGCACCAAATAGGCGCAGACGCGCTCCGACAGTTGCGCAAAAAGTGTGCAATGG	EMSA
Negative- P_{glnK}-F	CGGCGCTCGCGGGAGGGCCATACTGTCGGAGCGCGTCTCGCCCGGAGGCCGGCGTTCCG	EMSA
Negative- P_{glnK}-R	CGGAACGCCGGCCTCCGGGCGAGACGCGCTCCGACAGTATGGCCCTCCCGCGAGCGCCG	EMSA
exsA-F	CCAAGCTTCAAGGTACGACGGGAAGTGTTGG	ExsA cloning
exsA-R	CGAGCTCTCAATGATGATGATGATGATGGTTATTTTTAGCCCGGCATTC	ExsA cloning
NtrC-F	CGGAATTCATGAGCCGATCAGAGACCGTCTG	NtrC cloning
NtrC-R	CGGGATCCTTAGTGATGGTGATGGTGATGGTCGTCGCCTTCGTCGTC	NtrC cloning

GlnK-F	CGGAATTCATGAAGCTAGTCACTGCC	GlnK cloning
GlnK-R	CGGGATCCTTAGTGATGGTGATGGTGATGGATCGCGTCGGTATCGGTTTC	GlnK cloning
mini-*glnK*-F	CGAGCTCGCTGACGCAGGGGGCTTC	GlnK complement cloing

The restriction enzyme cleavage sites within the primers are underlined.

Table S3. Plasmids and strains used in this study.

Strain and plasmid	Description	Source
P. aeruginosa		
PA14	Wild type strain	This study
Δ*glnK*	PA14 *glnK* gene deletion mutant	This study
Δ*glnK*/att7::*glnK*	Δ*glnK* complementation with *glnK* inserted on chromosome, Tc^r	This study
Δ*ntrB*	PA14 *ntrB* gene deletion mutant	This study
Δ*ntrC*	PA14 *ntrC* gene deletion mutant	This study
Δ*glnK*Δ*ntrB*	PA14 *glnK* and *ntrB* genes double deletion mutant	This study
Δ*glnK*Δ*ntrC*	PA14 *glnK* and *ntrC* genes double deletion mutant	This study
glnK::Tn	PA14 *glnK* gene transposon insertion mutant	This study
glnK::Tn/att7:: *glnK*	*glnK*::Tn complementation with *glnK* inserted on chromosome, Tc^r	This study
Δ*exsA*	PA14 *exsA* gene deletion mutant	This study
E.coli		
DH5α	*F$^-$, φ80,lacZΔM15, Δ(lacZYA-argF)U169, deoR, recA1, endA1, hsdR17(r$_K^-$,m$_K^+$), phoA, supE44, λ$^-$, thi-1, gyrA96, relA1*	TransGen
S17-1	*recA*, *pro*, *hsdR*, RP4-2-Tc::Mu-Km::Tn7	Stratagene
BL21(DE3)	*F$^-$ ompT hsdSB (rB$^-$ mB$^-$) gal dcm (DE3)*	Solarbio
**Plasmids**		
pDN19-P$_{glnK}$-*lacZ*	pDN19lacΩ with *glnK* promoter; Tc^r	This study
pMMB67EH-*ntrC*-his	expression driven by an inducible tac promoter; Gm^r	This study
pMMB67EH-*glnK*-his	expression driven by an inducible tac promoter; Gm^r	This study
pMMB67EH-*gst*-his	expression driven by an inducible tac promoter; Gm^r	This study
pAK1900-*ntrB*-flag	expression driven by an inducible tac promoter; Ap^r	This study
pEx18Tc-Δ*ntrC*	*ntrC* gene deletion suicide plasmid; Tc^r	This study
pEx18Tc-Δ*glnK*	*glnK* gene deletion suicide plasmid; Tc^r	This study
pEx18Tc-Δ*ntrB*	*ntrB* gene deletion suicide plasmid; Tc^r	This study
pUC18T-mini-Tn7-Tc-	chromosomal integration of *glnK*-his，Tc^r	This study

glnK-his		
pTNS3	helper plasmid encoding the TnsABCD transposase subunits，Ap^r	[1]
pUCP20-*exsA*	*exsA* gene on the plasmid pUCP20, Ap^r	This study

References

1. Choi, K.H.; Schweizer, H.P. mini-Tn7 insertion in bacteria with single attTn7 sites: example Pseudomonas aeruginosa. Nat Protoc. 2006;1(1):153-61. doi: 10.1038/nprot.2006.24.
